# ESAT-6 protein suppresses allograft rejection by inducing CD4^+^Foxp3^+^ regulatory T cells through IκBα/cRel pathway

**DOI:** 10.3389/fimmu.2024.1529226

**Published:** 2025-01-09

**Authors:** Xiaofei Huang, Yuqun Zeng, Jingru Lin, Huazhen Liu, Chun-Ling Liang, Yuchao Chen, Feifei Qiu, Jonathan S. Bromberg, Zhenhua Dai

**Affiliations:** ^1^ Section of Immunology, The Second Affiliated Hospital of Guangzhou University of Chinese Medicine, Guangzhou, Guangdong, China; ^2^ Immunology Program, Guangdong Provincial Academy of Chinese Medical Sciences, Guangzhou, Guangdong, China; ^3^ Department of Nephrology, Zhejiang Provincial People’s Hospital, Affiliated People’s Hospital, Hangzhou Medical College, Hangzhou, Zhejiang, China; ^4^ Kidney and Pancreas Transplantation, Department of Surgery and Department of Microbiology and Immunology, University of Maryland School of Medicine, Baltimore, MD, United States

**Keywords:** transplantation, ESAT-6, alloimmunity, regulatory T cell (Treg), immunoregulation

## Abstract

**Background:**

Maintenance immunosuppression is required for suppression of alloimmunity or allograft rejection. However, continuous use of immunosuppressants may lead to various side effects, necessitating the use of alternative immunosuppressive drugs. The early secreted antigenic target of 6 kDa (ESAT-6) is a virulence factor and immunoregulatory protein of mycobacterium tuberculosis (Mtb), which alters host immunity through dually regulating development or activation of various immune cells. ESAT-6 may be a potential alternative immunosuppressant that could be utilized to suppress allograft rejection although it remains unknown whether ESAT-6 actually regulates alloimmunity.

**Methods:**

In this study, murine skin or heart allotransplantation was performed to determine the effects of ESAT-6 protein on allograft survival. Flow cytometric analyses were conducted to quantify CD4^+^Foxp3^+^ Tregs, while immunohistochemistry was carried out to observe allograft immunopathology. Western blotting was used to detect IĸBα/c-Rel signaling during Treg induction. Finally, CD4^+^CD25^-^ conventional T cells were cultured to induce Tregs and their proliferation.

**Results:**

Here we found that ESAT-6 significantly extended murine skin and heart allograft survival, alleviated CD3^+^ T cell infiltration and increased Foxp3^+^ Tregs in an allograft. ESAT-6 augmented the percentage of CD4^+^Foxp3^+^ Tregs, whereas it decreased the frequency of Th1 and CD4^+^/CD8^+^ effector T cells in spleen and lymph nodes (LNs) posttransplantation. ESAT-6 also induced CD4^+^Foxp3^+^ Tregs from CD4^+^CD25^-^ T cells *in vitro* by activating IĸBα/c-Rel signaling pathway, whereas inhibition of c-Rel signaling blocked Treg induction. Moreover, it suppressed conventional CD4^+^CD25^-^ T cell proliferation *in vitro* in the absence of antigen-presenting cells (APCs), with an increase in IL-10 and decrease in IFN-γ production. On the other hand, it did not significantly alter DC maturation after allotransplantation.

**Conclusion:**

Thus, ESAT-6 suppresses alloimmunity and inhibits allograft rejection by inducing CD4^+^Foxp3^+^ Tregs through IĸBα/c-Rel signaling pathway.

## Introduction

Organ transplantation is the last treatment option for some patients with terminal organ failure, such as heart and liver failure. The half-life of a grafted organ, nevertheless, is far from being desirable, mainly due to immune-mediated rejection, especially chronic rejection, following allotransplantation (1). Although various immunosuppressive agents, including steroids, calcineurin inhibitors (tacrolimus), antimetabolites (mycophenolate) and inhibitors of mammalian target of rapamycin (sirolimus) can suppress transplant rejection, they may also cause various side effects, such as renal dysfunction, infections and hyperlipidemia (2–6). Thus, developing new drugs with minimal toxicity is necessary although immunosuppressants currently available can meet the basic demand in clinic.

The early secreted antigenic target of 6 kDa (ESAT-6) is a small protein of 95 amino acids secreted by Mycobacterium tuberculosis (Mtb). ESAT-6 is only detected in clinical isolates of Mtb and virulent Mycobacterium bovis (M. bovis), whereas it has not been found in attenuated sub-strains of M. bovis BCG (7, 8). As a virulence determinant of Mtb (9), ESAT-6 has been well studied to elucidate the mechanisms underlying its role in Mtb pathogenesis (10–14). Although ESAT-6, as an antigenic molecule of Mtb, plays redundant roles in regulating activation/function of many immune cells (14, 15), recent studies have highlighted its inhibitory effects on some of the immune cells. For instance, ESAT-6 has been shown to inhibit MyD88/NFkB signaling in macrophages by binding to their TLR2 (16). ESAT-6 attenuated enzyme activity of the matrix metalloproteinase-9 and inhibited COX-2 and inducible nitric oxide synthetase (iNOS) in RAW 264.7 macrophages stimulated by LPS (17). It also suppressed the expression of proinflammatory cytokines in macrophages through modulating miR-222-3p (18). More importantly, it can suppress T cell activation *in vitro (* 19). However, it remains unknown if ESAT-6 inhibits alloimmunity or allograft rejection. The rationale for determining effects of ESAT-6 on transplant rejection is that it inhibits the function of both macrophages and T cells.

In this study, we determined the effects of ESAT-6 on allograft rejection and the potential mechanisms underlying its effects on alloimmunity. Our data demonstrated that ESAT-6 extended survival time of both skin and heart allografts in mice. Combined treatment with both ESAT-6 and rapamycin further prolonged allograft survival. ESAT-6 reduced CD3^+^ T cell infiltration while increasing Foxp3^+^ Treg numbers in the skin allograft. ESAT-6 augmented the frequency of CD4^+^Foxp3^+^ Tregs, whereas it reduced that of Th1 and CD4^+^/CD8^+^ effector T cells in both LNs and spleen of recipient mice. Furthermore, it induced CD4^+^Foxp3^+^ Tregs from CD4^+^CD25^-^ T cells *in vitro* through acting on their IĸBα/c-Rel pathway. Thus, ESAT-6 indeed modulates alloimmunity in mice.

## Materials and methods

### Animals and protein

BALB/c and C57BL/6 mice (male, 7-8 weeks-old) were purchased from Guangdong Medical Laboratory Animal Center (Guangzhou, Guangdong Province, China). Mice were housed and maintained under a specific pathogen-free (SPF) condition. The animal protocols performed in this study were approved by the Animal Ethics Committee of Guangdong Provincial Academy of Chinese Medical Sciences (Approval No. 2020091).

ESAT-6 was purchased from China Peptides Co. Ltd. Briefly, ESAT-6 gene was obtained by gene synthesis and cloned into plasmid pET32a containing c-myc tag, which then was transfected into E.coli. Subsequently, ESAT-6 protein was expressed, purified and verified through SDS-PAGE (purity>95%), followed by endotoxin removal using the Protein Endotoxin Removal Kits (Beyotime, China, endotoxin <0.1 EU/ml).

### Treatment of mice

Most recipient mice were randomly divided into four groups, including control (vehicle for ESAT-6), rapamycin (Rapa, MCE, USA), ESAT-6 (China Peptides Co. Ltd., China) and ESAT-6 plus Rapa groups. In some experiments, the culture filtrate protein (CFP-10, China Peptides Co. Ltd.) was also used as another control group. Rapa (1 mg/kg) was administered *i.p*. daily for three weeks or until allograft rejection, whichever came earlier, while ESAT-6 or CFP-10 (50μg/kg) was injected *i.p*. on days 0, 2, 4, 6 and 8 following allotransplantation. Both ESAT-6/CFP-10 and rapamycin were dissolved in saline. To deplete Tregs, recipient mice were administered with anti-CD25 mAb (Clone PC61.5, Bio X Cell, USA) at 0.1 mg on days 0, 3 and 7.

### Skin transplantation

Wild-type (WT) C57BL/6 mice received skin grafts from BALB/c donor mice. Skin allotransplantation was performed as described in previous studies (20, 21). Briefly, full-thickness trunk skin sized approximately 1 cm^2^ was grafted onto the right dorsal flank of a recipient and covered with a sterile bandage (Johnson & Johnson). The skin graft was monitored daily after removal of the bandage at eight days posttransplantation. Skin allograft rejection was defined as graft necrosis of larger than 90% of the graft area.

### Heterotopic heart transplantation

Cervical heterotopic heart transplantation in recipient mice was performed. Briefly, the right cervical common carotid artery and the external jugular vein of recipients were incised, with vascular cuffs being made by 24-G and 22-G intravenous catheters (BD Biosciences), respectively, and connected to the ascending aorta and the pulmonary artery of a donor heart (22). Survival of heart allograft was monitored by visual observation and palpation, while allograft rejection was defined as the cessation of cardiac contractions.

### Histological analysis

Skin allografts were fixed in 4% paraformaldehyde for 18 hours and then embedded in paraffin after dehydration. Paraffin slides with 3-μm thickness were made, deparaffinized and stained with hematoxylin and eosin (H&E staining). For immunohistochemistry, sections were incubated with primary anti-CD3 (clone: E4T1B, 1:1000; CST) or ani-Foxp3 (clone: D608R, 1:1000; CST) Ab at 4˚C overnight, and then with secondary Ab HRP–anti-rabbit IgG (Maxim) at room temperature for 30 min. Finally, the slides were colored with diaminobenzidine (DAB, Maxim). For quantitative analysis, slides for H&E were imaged at a magnification of 100× and those for IHC were 200x. The cellular infiltration and integrated optical density (IOD) of CD3^+^ or Foxp3^+^ staining in the images were measured using software ImageJ.

### Flow cytometric analysis

Draining lymph node and spleen cells were harvested and stained with anti-CD4-FITC or APC (clone: RM4.5), CD8-FITC or PE (clone: 53-6.7), CD44-PerCP-Cyanine5.5 (clone: IM7), CD62L-PE-Cy7 (clone: MEL-14), CD11C-PE (clone: HL3), CD80-FITC (clone: HL3), CD86-APC (clone: GL-1), IFN-γ-APC (clone: XMG1.2), IL-17-PE (clone: TC11-18H10) and FoxP3-PE (clone: FJK-16s) mAbs (eBioscience or BD Biosciences). To analyze intracellular IFN-γ, cells were cultured with PMA and ionomycin in the presence of monensin for six hours before the intracellular staining. To stain for intracellular FoxP3 and IFN-γ, cells were fixed, permeated using the Foxp3/Transcription Factor Fixation/Permeabilization Concentrate and Diluent Kits (eBioscience), stained with anti-FoxP3 or anti-IFN-γ Ab, and analyzed using FACSAria III (BD Biosciences). To purify CD4^+^CD25^-^ conventional T cells for Treg induction *in vitro*, splenocytes were stained with anti-CD4 PerCP-Cyanine5.5 (clone: RM4-5, eBioscience, USA) and anti-CD25-PE Abs (clone: 3C7, eBioscience, USA), and then CD4^+^CD25^-^ T cells were sorted using FACSAria III (BD Biosciences). The purity of the sorted cells was typically >97%.

### Induction of Tregs and measurement of T cell proliferation *in vitro*

FACS-sorted CD4^+^CD25^-^ T cells (2 x 10^6^/ml) were cultured in the complete RIPM 1640 medium (10%FBS, 2mM glutamine, 100 U/ml penicillin and 100 µg/ml streptomycin) and stimulated with anti-CD3/anti-CD28 Abs (5 μg/ml) in the presence of rIL-2 (2ng/ml, Peprotech) for four days. Cells were also treated with ESAT-6 (0.5, 1 and 2 μg/ml, respectively), CFP-10 (1 μg/ml), rapamycin (0.1μM), or E/Z-IT-603 (an inhibitor of IĸBα/c-Rel signaling, 10 μM) in some groups. Tregs then were quantified using flow cytometry. To measure conventional T cell proliferation in a relatively physiological setting, primarily nylon wood-enriched T cells were stained with CFSE (5 μM) at room temperature for 15 min, washed and cultured for four days, and finally analyzed through a flow cytometer (NovoCyte Quanteo, Agilent, USA).

### Measurement of IFN-γ and IL-10 by ELISA

The proteins of IFN-γ and IL-10 in the supernatant of the culture were measured using ELISA kits according to the manufacturer’s instructions (Boster, China), while the absorbance was read at 450 nm in a microplate spectrophotometer (Thermo Fisher Scientific, USA).

### Western blotting

Whole protein was obtained from RIPA buffer after lysing cells, while the nuclear protein of the cells was extracted by the Nuclear and Cytoplasmic Protein Extraction Kits (Beyotime, China). The concentration of protein sample was measured using a BCA protein assay kit (Thermo Fisher Scientific, USA). Proteins were run in 10% SDS-PAGE gels and transferred to a PVDF-membrane. After blocking in Tris-buffered saline with Tween-20 containing 5% (w/v) BSA at room temperature for 1-h, the membrane was incubated with rabbit primary Abs, including anti–p-IKBα (clone: 14D4, 1:1000, CST), anti-IKBα (clone: 44D4, 1:1000, CST), anti-α-tubulin (clone: 11H10, 1:1000; CST), anti–c-Rel (clone: EPR25178-58, 1:1000, Abcam) and anti-Histone H3 (clone: AFB7534, 1:3000, Affinity) Abs, at 4˚C overnight. After incubation, the membrane was washed with TBST and then incubated with a secondary antibody, HRP-conjugated goat anti-rabbit IgG or anti-mouse IgG (1:2000, CST, USA) for one hour. Blot signals were detected by a Bio-Rad Gel imaging system and analyzed with ImageJ software.

### Statistical analysis

Statistical comparisons of the means were performed using one-way ANOVA for multiple groups or Student *t*-test for two groups only. Data were analyzed using GraphPad Prism 8. The analysis of graft survival was performed using Kaplan–Meier method (log-rank test). A value of P < 0.05 was considered statistically significant.

## Results

### ESAT-6 extends murine skin allograft survival while increasing Foxp3^+^ Tregs in a skin allograft

**Figure 1:**
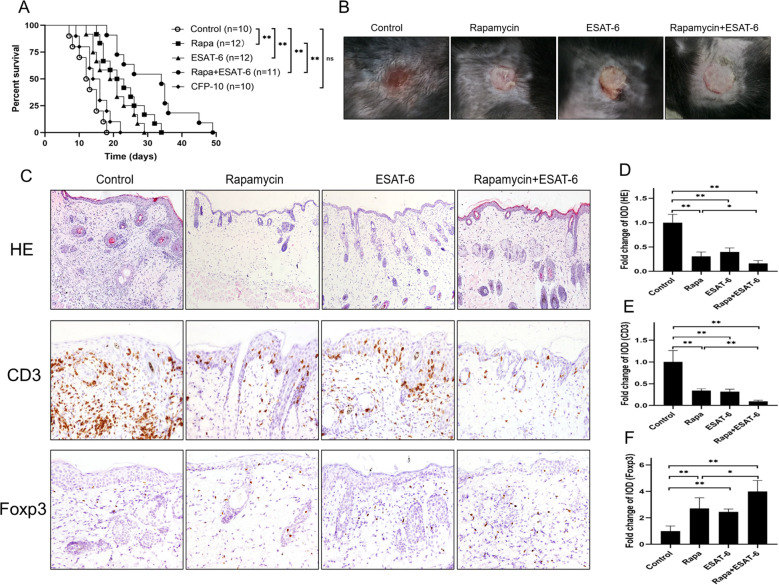
ESAT6 prolongs murine skin allograft survival and increases Foxp3^+^ Treg cells in allografts. C57BL/6 mice were transplanted with BALB/c skin and administrated with rapamycin (Rapa) and/or ESAT-6. **(A)** Skin allograft survival was observed with Kaplan–Meier curves plotted (*p <0.05, **p < 0.01, n = 10-12). **(B)** Representative of skin allografts 10 days after transplantation. **(C)** H&E staining and IHC for CD3 and Foxp3 on skin allografts in recipient mice 10 days after transplantation. **(D)** The area of cellular infiltration in an allograft was quantified as integrated optical density (IOD) for HE-staining. **(E, F)** The IOD of CD3^+^ and Foxp3^+^ Treg cells in the skin was calculated using ImagePro plus. Values were shown as fold change relative to control group that was set as 1.0. Data are presented as the mean ± SD (n = 6 grafts/group). One of three separate experiments is shown.

ESAT-6 has been shown to regulate immune responsiveness. To determine the effects of ESAT-6 protein on allograft survival, C57BL/6 mice were transplanted with skin derived from BALB/c mice and treated with ESAT-6, culture filtrate protein-10 (CFP-10), rapamycin (Rapa) or ESAT-6 plus Rapa. As shown in **Figure 1A**, ESAT-6 prolonged skin allograft survival in recipient mice compared to the control, with a statistical significance (median survival time: MST = 20 vs. 12 days). As a positive control, Rapa also prolonged the allograft survival (MST = 22 vs. 12). Moreover, the combined treatment with ESAT-6 and Rapa further extended allograft survival compared to the treatment with either ESAT-6 alone (MST = 34 vs. 20) or Rapa alone (MST = 34 vs. 22 days). However, CFP-10 did not significantly alter allograft survival time. Like ESAT-6, CFP-10 is a protein also expressed in RD1 region of Mtb and thus can serve as a control for ESAT-6. Taken together, our findings suggest that ESAT-6 significantly suppresses allograft rejection.

We then asked if ESAT-6 could diminish cellular infiltration in a transplant, as determined by H&E and IHC stainings after allotransplantation. Representatives of rejected or accepted skin transplants are shown in **Figure 1B**. H&E staining displayed marked cellular infiltration in untreated and transplanted control mice, while much less cellular infiltration was found in recipient mice treated with ESAT-6 or Rapa (**Figures 1C, D**). Treatment with both ESAT6 and Rapa further suppressed cellular infiltration compared to Rapa alone (**Figures 1C, D**). Similarly, ESAT-6 or Rapa also inhibited CD3^+^ T cell infiltration in allografts compared with the control group (**Figure 1E**), while a further decrease in CD3^+^ T cells was observed in the group treated with ESAT6 plus Rapa compared to Rapa alone.

CD4^+^Foxp3^+^ Tregs are essential for allograft survival or immune tolerance (23, 24). Therefore, we further analyzed Foxp3 expression in a skin allograft through IHC staining. We found that either ESAT-6 or Rapa enhanced Foxp3 expression in skin transplants (**Figure 1F**). Importantly, treatment with ESAT-6 plus Rapa induced even more Foxp3 expression in an allograft compared to that with Rapa treatment alone.

### ESAT-6 also extends cardiac allograft survival by increasing CD4^+^Foxp3^+^ Tregs, while depleting Tregs reversed ESAT-6-mediated extension of cardiac allograft survival

**Figure 2:**
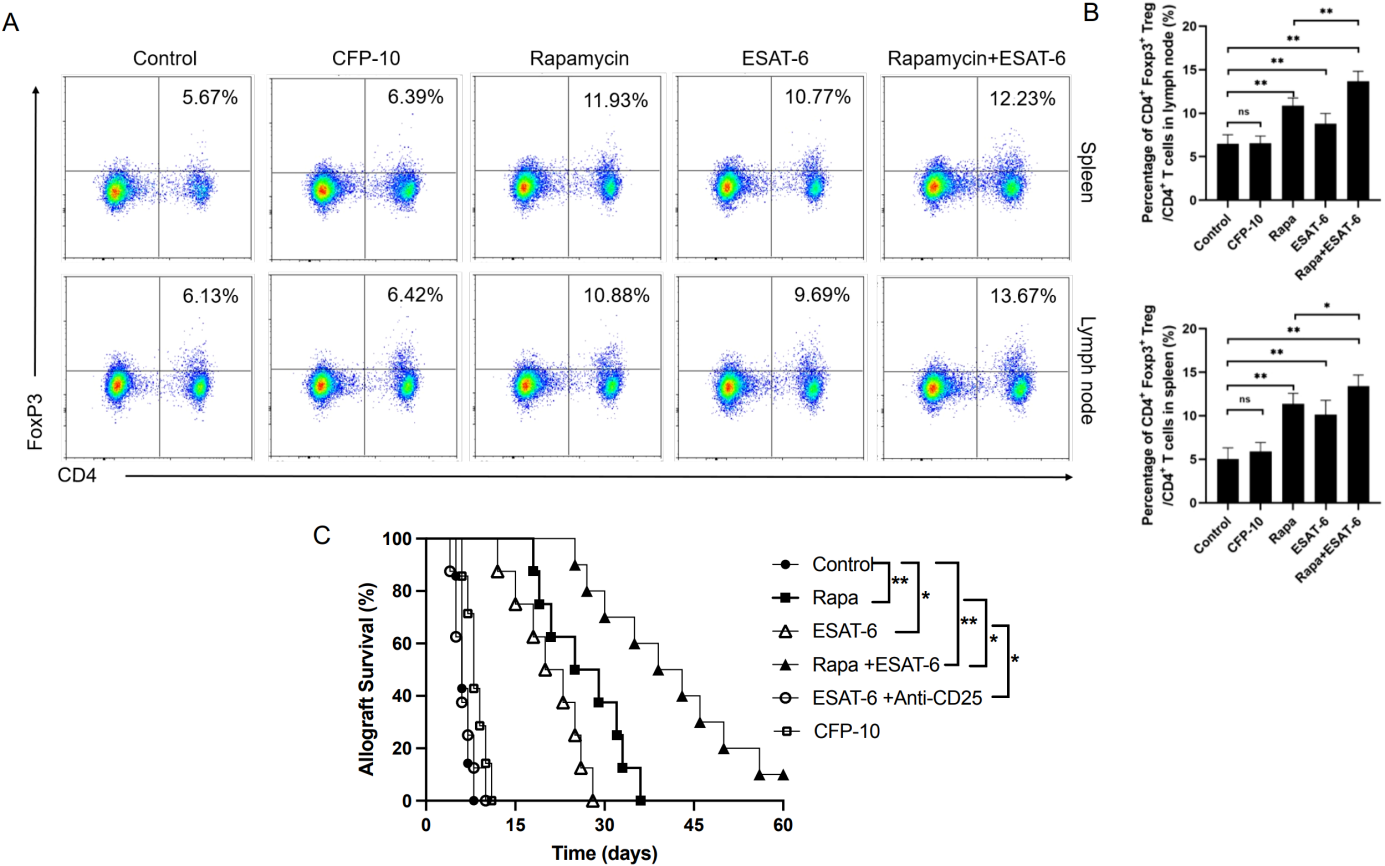
ESAT-6 extends cardiac allograft survival by increasing CD4^+^Foxp3^+^ Tregs, while depleting Tregs reverses ESAT-6-mediated allograft survival. C57BL/6 mice were transplanted with a BALB/c heart and treated with Rapa, CFP-10 and/or ESAT-6. Some recipients were treated with depleting anti-CD25 Ab to deplete Tregs. Spleen and draining LN cells were isolated seven days post-transplantation, while CD4^+^Foxp3^+^ Tregs were quantified by FACS. **(A)** Shown are the representative dot plots of CD4^+^Foxp3^+^ Tregs in the spleen and LNs. **(B)** Column graphs present the frequencies of Tregs in spleen and LNs. Data are shown as mean ± SD (n = 4 mice/group per experiment, *p < 0.05 and **p < 0.01). One of three separate experiments is shown. **(C)** Cardiac allograft rejection was observed daily (n= 7-10 mice/group accumulated).

C57BL/6 mice were transplanted with a BALB/c heart and treated with Rapa and/or ESAT-6. Spleen and draining cervical LN cells were isolated seven days posttransplantation, while CD4^+^Foxp3^+^ Tregs were quantified by flow cytometric analysis. Treatment with ESAT-6 or Rapa alone increased the percentages of CD4^+^Foxp3^+^ Tregs in the spleen and LNs (**Figures 2A, B**) compared to the control group, whereas CFP-10 failed to do so. Importantly, the treatment with ESAT-6 plus Rapa further increased the frequency of Tregs compared to Rapa alone. On the other hand, cardiac allograft survival was significantly prolonged by ESAT-6 (MST= 21 vs. 6 days) or Rapa alone (MST= 27 vs. 6 days), while combined treatment further extended allograft survival compared to Rapa alone (MST= 41 vs. 27 days) (**Figure 2C**). However, treatment with depleting anti-CD25 Ab reversed the EAST-6-mediated extension of allograft survival (**Figure 2C**). Our findings suggest that ESAT-6 also prolongs cardiac allograft survival by increasing CD4^+^Foxp3^+^ Tregs.

### ESAT-6 reduces the frequency of Th1 cells in recipient mice

**Figure 3:**
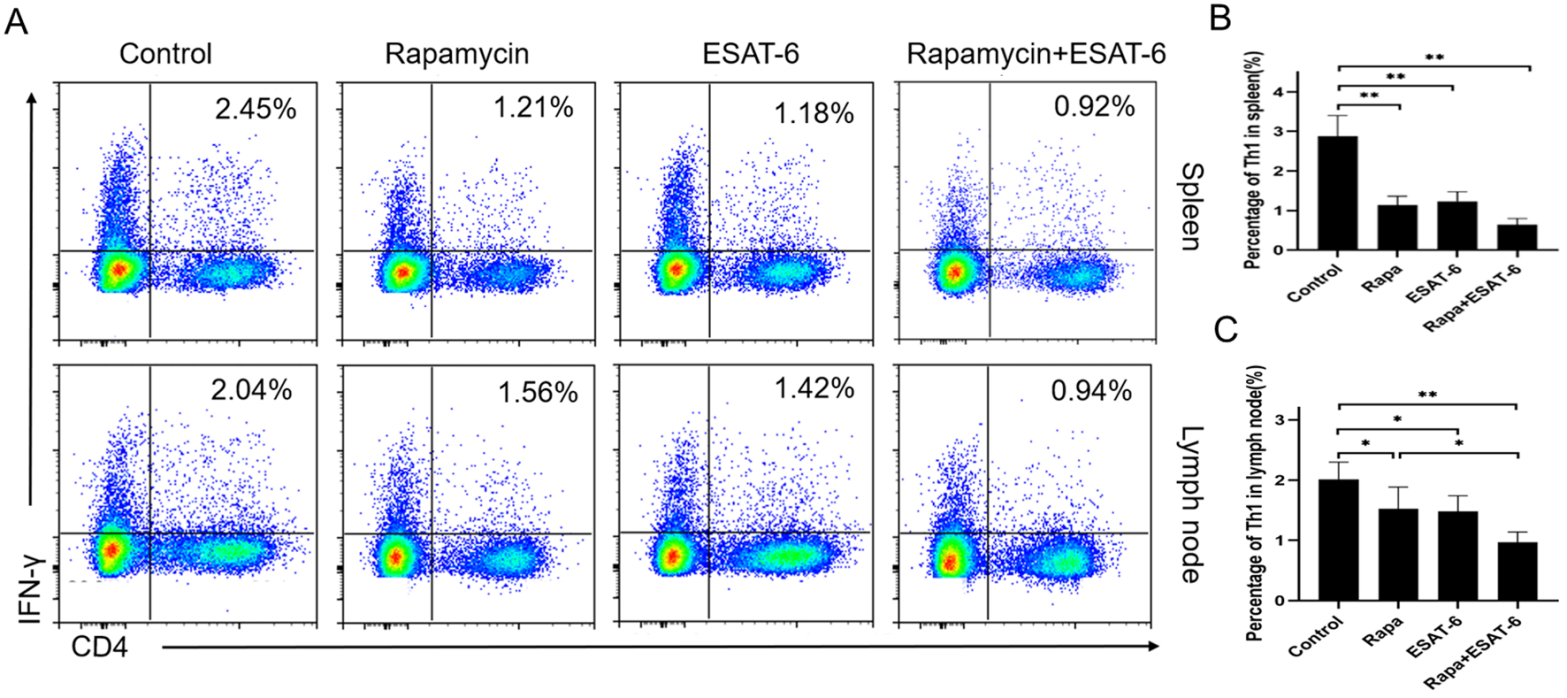
ESAT6 reduces the frequency of Th1 cells *in vivo*. Draining lymph node (LN) and spleen cells from ESAT-6- or Rapa-treated C57BL/6 recipient mice receiving BALB/c skin were isolated 10 days after transplantation and analyzed through FACS. Shown are the representative dot plots of CD4^+^IFN-γ^+^ T cells (Th1) in the spleen and LNs **(A)**. Column graphs display the frequency of Th1 cells in spleen **(B)** and LNs **(C)**. Data of column graphs are shown as the mean ± SD (n = 5-6 mice/group). Shown is one of the two separate experiments.

T helper 1 (Th1) cells play a pivotal role in mediating alloimmune responses or allograft rejection. Since we found that ESAT-6 alleviated CD3^+^ T cell infiltration in a skin allograft, we then assessed if it regulated the Th1 response *in vivo*. Recipient mice receiving a skin allograft were treated with ESAT-6 and/or Rapa, and then draining lymph node (LN) and spleen cells from recipient mice were harvested and analyzed by FACS 10 days after transplantation. As shown in **Figure 3**, either ESAT-6 or Rapa reduced the frequency of CD4^+^IFN-γ^+^ Th1 cells in both spleens (**Figures 3A, B**) and LNs (**Figures 3A, C**) of recipient mice. The percentage of Th1 cells in LNs of recipients was further decreased by treatment with both ESAT-6 and Rapa (**Figure 3C**). These results suggest that ESAT-6 indeed inhibits Th1 differentiation after allotransplantation.

Since activation and maturation of DCs are important for T cell differentiation/activation, we then examined whether ESAT-6 would affect the maturation of DCs ten days after skin transplantation. Using flow cytometric analyses, we found that ESAT-6 did not significantly alter the frequency of CD11c^+^CD86^+^ or CD11c^+^CD80^+^ cells in the spleen of recipient mice (**Supplementary Figure S1**), indicating that the maturation of DCs, unlike Th1 differentiation, is not particularly affected by the treatment with ESAT-6 in the face of vigorous alloimmune responses.

### ESAT-6 lowers the percentage of CD4^+^ and CD8^+^ effector T Cells after allotransplantation

**Figure 4:**
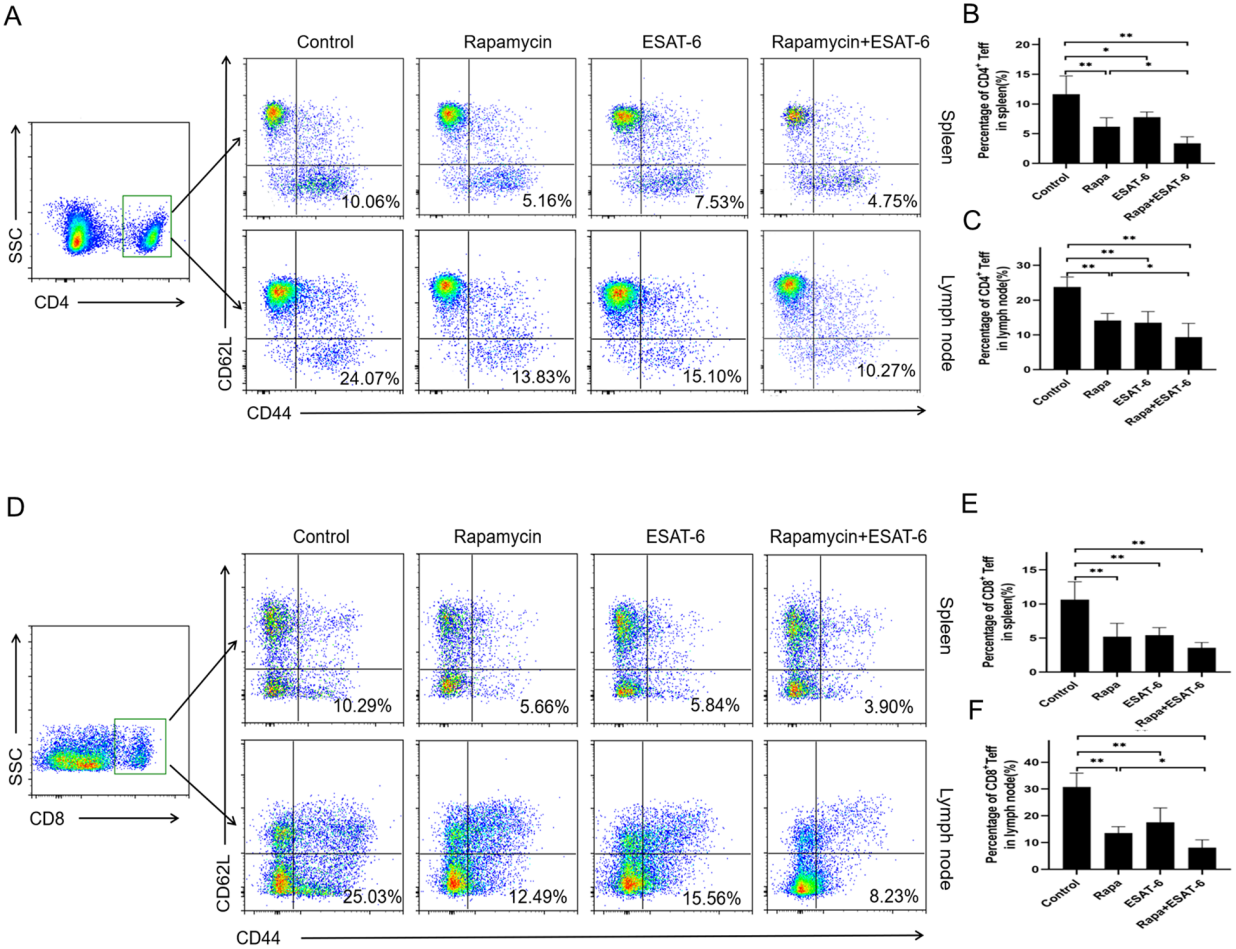
ESAT-6 reduces the frequency of CD4^+^ and CD8^+^ effector T cells *in vivo*. LN and spleen cells from recipient mice treated with ESAT-6 and/or Rapa were harvested 10 days after transplantation and analyzed by FACS. Shown are the representative dot plots of CD4^+^CD44^high^CD62L^low^ T cells (Teff) in LNs and spleen of recipient mice **(A)**. Column graphs display the frequency of CD4^+^ Teff in spleen **(B)** and LNs **(C)**. Also shown are the representative dot plots of CD8^+^CD44^high^CD62L^low^ in spleen and LNs **(D)**. Column graphs demonstrate the frequency of CD8^+^ Teff in spleen **(E)** and LNs **(F)**. Data of column graphs are shown as the mean ± SD (n = 4-5 mice/group per experiment). One of the two separate experiments is shown.

We further asked whether it would reduce effector CD4^+^ and CD8^+^ T cells *in vivo*. Draining LN and spleen cells from recipient mice receiving skin allografts were isolated 10 days posttransplantation, and the frequency of CD44^high^CD62L^low^ effector CD4^+^ and CD8^+^ T cells were analyzed by FACS. Compared to control group, ESAT-6 or Rapa alone reduced the frequency of CD4^+^CD44^high^CD62L^low^ (**Figures 4A–C**) and CD8^+^D44^high^CD62L^low^ (**Figures 4D–F**) effector T cells (Teff) in both spleen and LNs of the recipient mice. Moreover, compared to the treatment with Rapa alone, combined treatment with both ESAT-6 and Rapa further decreased the percentage of CD4^+^CD44^high^CD 62L^low^ Teff in both spleen and LNs (**Figures 4B, C**) as well as the percentage of CD8^+^CD44^high^CD62L^low^ Teff in LNs only (**Figure 4F**). These results indicate that ESAT-6 inhibits the generation of both CD4^+^ and CD8^+^ effector T cells in the context of allotransplantation.

### IĸBα/c-Rel signaling-dependent induction of Tregs *in vitro* by ESAT-6

**Figure 5:**
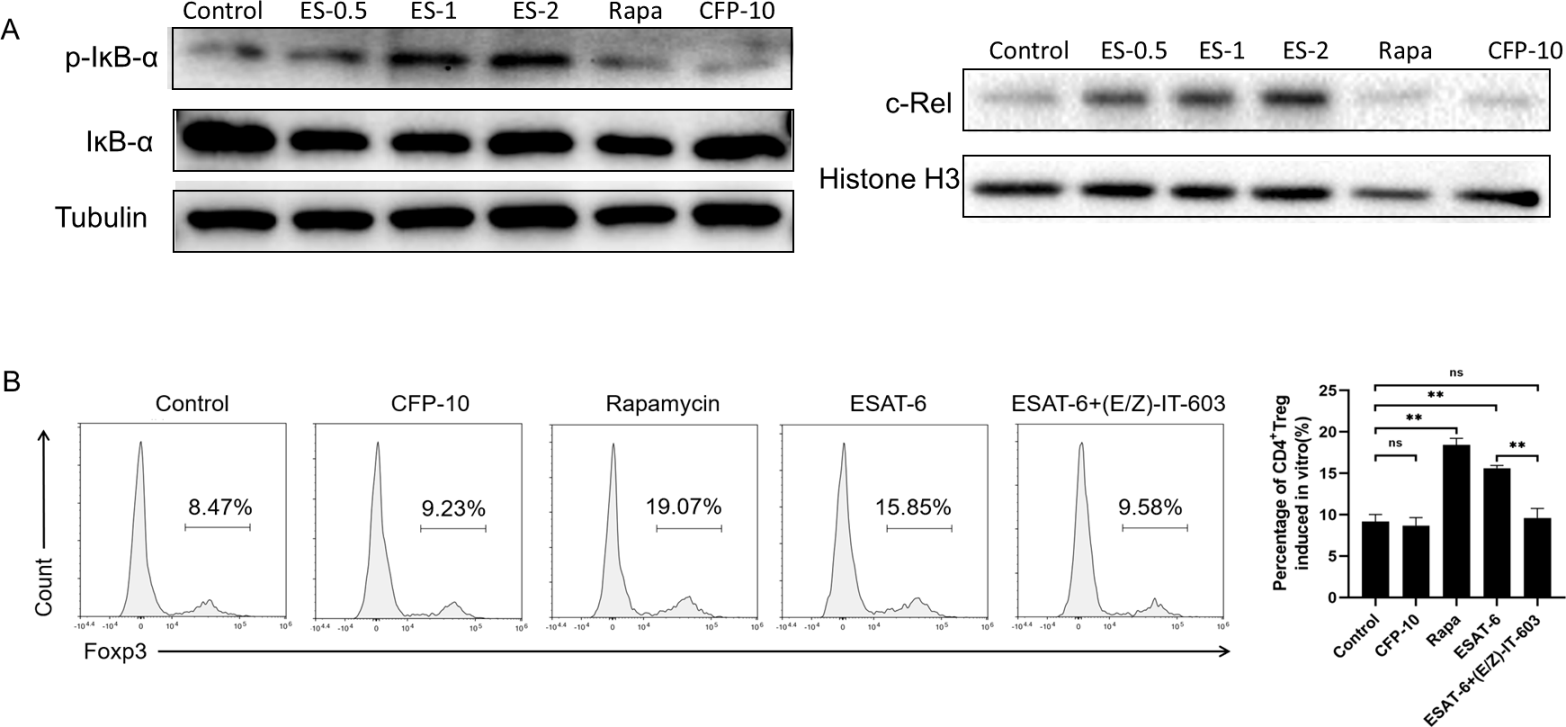
ESAT-6 induces Tregs *in vitro* by activating IĸBα/c-Rel signaling in T cells. FACS-sorted CD4^+^CD25^-^ T cells were stimulated with anti-CD3/CD28 Abs plus IL-2 in the absence or presence of ESAT-6, CFP-10, Rapa, or E/Z-IT-603 (an inhibitor of IĸBα/c-Rel) for 2 (IĸBα/c-Rel signaling) or 4 days (Treg induction). **(A)** IĸBα/c-Rel signaling was detected using Western blotting. Shown are representatives of the images displaying IĸB-α, p-IĸB-α, tubulin, c-Rel and histone H3 expressions in T cells. Tubulin and histone H3 were used as a loading control (ES: ESAT-6, ES-0.5 = 0.5μg/ml, ES-1 = 1μg/ml, and ES-2 = 2μg/ml). **(B)** Cells were also analyzed via flow cytometry to measure FoxP3^+^ Treg induction *in vitro*. (E/Z-IT-603: a c-Rel inhibitor). Data of column graphs are shown as the means ± SD (n = 4/group per experiment, **p<0.01). One of the two separate experiments is shown.

The c-Rel in NF-ĸB pathway is critical for the transcriptional regulation of expression of forkhead box P3 (FoxP3), which governs the Treg development (25). Since we found that ESAT-6 increased CD4^+^Foxp3^+^ Tregs, we hypothesized ESAT-6 might promote Treg development by acting on IĸBα/c-Rel signaling in CD4^+^ T cells. The proteins of IκBα, phospho-IκBα and c-Rel isolated from conventional CD4^+^CD25^-^ T cells, which were cultured and stimulated by anti-CD3/CD28 Abs for two days, were measured *via* Western blotting. As shown in **Figure 5A**, ESAT-6 augmented phosphorylation of IκBα and promoted nuclear c-Rel accumulation, seemingly in a dose-dependent manner (ES: 0.5, 1, 2 μg/ml), compared to the control group. As controls, neither Rapa nor CFP-10, which is a chaperone protein of ESAT-6 and also important for Mtb virulence (26), enhanced c-Rel expression in the nucleus. These findings indicate that ESAT-6, but not CFP-10, enhances IĸBα/c-Rel signaling in conventional CD4^+^CD25^-^ T cells, resulting in generation of more Tregs.

To further confirm that ESAT-6 promotes Treg development by acting on c-Rel signaling, FACS-sorted CD4^+^CD25^-^ conventional T cells were cultured with anti-CD3/anti-CD28 Abs in the absence or presence of ESAT-6 (1 μg/ml) for four days. In some groups, cells were treated with ESAT-6 plus E/Z-IT-603 that blocks c-Rel signaling. What we found was that ESAT-6 induced CD4^+^Foxp3^+^ Tregs *in vitro*, and so did Rapa (**Figure 5B**) as a positive control, while blocking c-Rel with E/Z-IT-603 reversed the effects of ESAT-6 on the Treg generation.

### ESAT-6 suppresses the proliferation of conventional T cells and their production of IFN-γ *in vitro*

**Figure 6:**
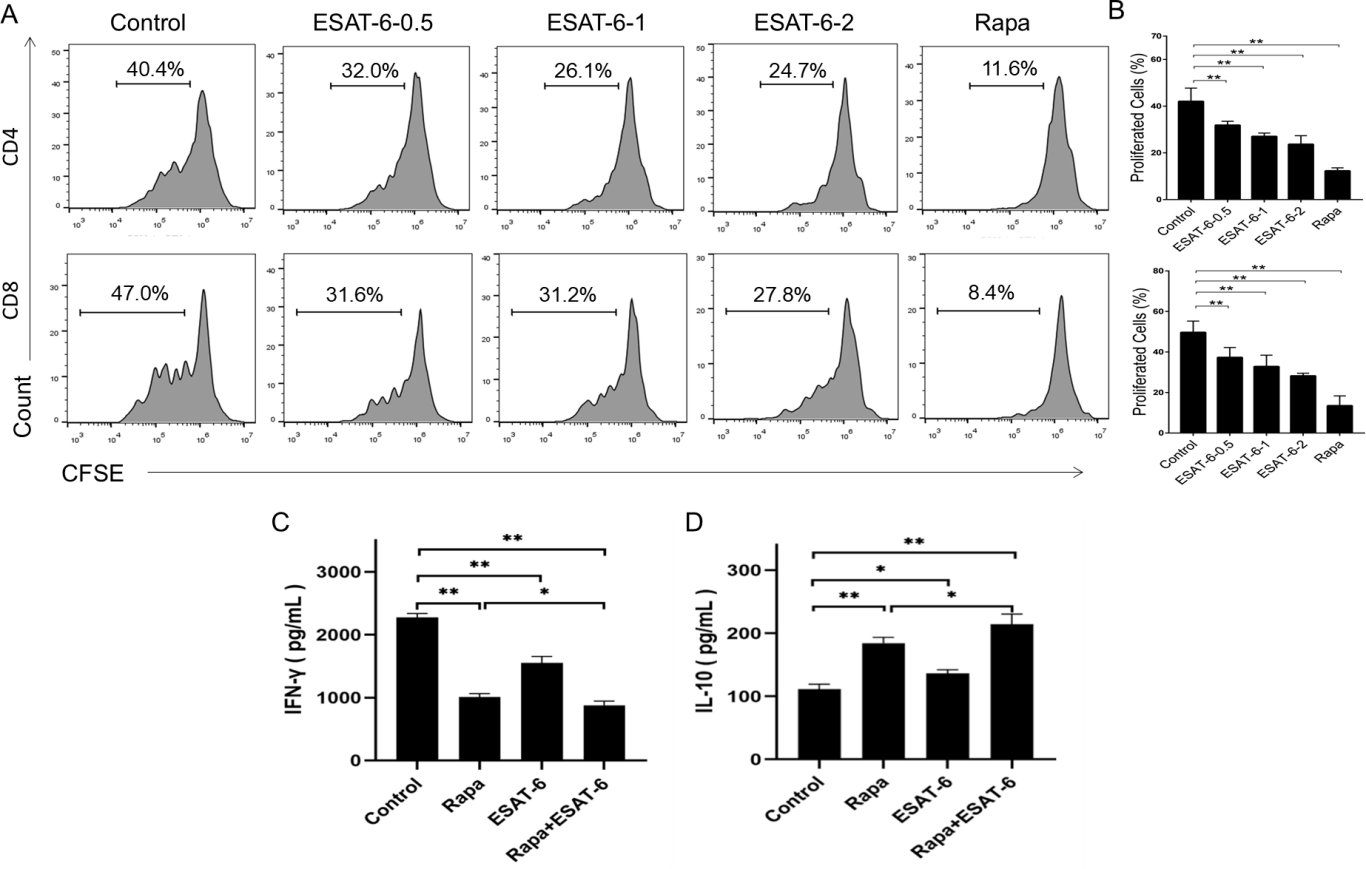
ESAT-6 suppresses conventional T cell proliferation and their cytokine secretion *in vitro*. **(A, B)** Nylon wood-enriched T cells were stained with CFSE and then stimulated with anti-CD3/anti-CD28 Abs in the absence or presence of Rapa and/or ESAT-6 for four days. Shown are CD4^+^/CD8^+^ T cell population that underwent proliferation, as represented by the dilution of CFSE. **(C, D)** The levels of IFN-γ and IL-10 in the supernatant of T cell culture were determined by ELISA after 4 days’ culture. For ELISA assays, cells were cultured without CFSE staining, but with ESAT-6 and/or Rapa. Data are shown as the means ± SD (n = 3-4/group, *p <0.05, **p<0.01). One of the two separate experiments is shown here.

To determine an effect of ESAT-6 on conventional T cell activation or function, nylon wood-enriched T cells from splenocytes were stained with CFSE and then cultured with anti-CD3/anti-CD28 Abs in the presence of ESAT-6 (0.5, 1, 2 μg/ml, respectively) for four days. As shown in **Figures 6A, B**, either ESAT-6 or Rapa significantly inhibited both CD4^+^ and CD8^+^ T cell proliferation *in vitro*. We also found that either ESAT-6 or Rapa inhibited IFN-γ secretion (**Figure 6C**) while increasing IL-10 secretion (**Figure 6D**) in the culture of T cells. Joint treatment with both ESAT-6 and Rapa resulted in a further decrease in IFN-γ and increase in IL-10 production compared to ESAT-6 or Rapa treatment alone. These data suggest that ESAT-6 inhibits conventional T cell proliferation and activation.

## Discussion

ESAT-6 was originally described as an immunogenic protein and virulence factor of Mtb (9, 27, 28), and much work has been done on the diagnostic methods and vaccine development involving ESAT-6. Recently, ESAT-6 has also been reported to regulate both innate and adaptive immunity (16, 19, 29). Nevertheless, the roles of ESAT6 in an immune response to an allograft after transplantation and its mechanisms of action remain unknown and need to be elucidated. Given that ESAT-6 could suppress T cell activation (19), we then sought to determine the effects of ESAT-6 on T cells in the context of allotransplantation and allograft rejection. We found that ESAT-6 suppressed allograft rejection by inducing CD4^+^FoxP3^+^ Tregs through activating their IĸBα/c-Rel pathway. This finding may have implications for clinical transplantation since ESAT-6 has been proved to be safe without any major side-effect (30).

Previous studies have shown that ESAT-6 suppresses the proliferation of Mtb-responsive human T cells and their production of IFNγ, thus inhibiting the Th1 response (19, 31). Others demonstrated that some Ag-specific T cells could respond to EAST-6 antigen itself (32–34), although ESAT-6-specific T cells were more terminally differentiated than those specific for other immunodominant antigens of Mtb (35). Using a murine model of skin and heart allotransplantation, we have presented the clear evidence that ESAT-6 is also effective in the suppression of allograft rejection. ESAT-6 extended allograft survival and alleviated the histopathological severity of cellular rejection. The therapeutic effects of ESAT-6 on allograft rejection were associated with a decrease in Th1 and CD4^+^/CD8^+^ effector T cells and an increase in Treg numbers.

It is generally acknowledged that T cell-mediated immune response plays an essential role in the occurrence and development of allograft rejection. IFN-γ is one of the main proinflammatory cytokines released by Th1 and other types of cells. Suppression of Th1 cells or cytokines can effectively alleviate allograft rejection (36). In this study, we demonstrated that ESAT-6 significantly reduced CD4^+^IFN-γ^+^ Th1 cells in recipient mice and the IFN-γ level in the supernatant of cultured T cells. We also found that ESAT-6 suppressed T cell proliferation *in vitro*. These data are consistent with previously published studies showing that ESAT-6 impeded the production of IFN-γ in human T cells stimulated by either Mtb or the combination of anti-CD3 and anti-CD28 Abs (19, 31).

Regulatory T (Treg) cells are an immunosuppressive minor subset of T cells, which are attractive candidates for treating autoimmune diseases, allergic diseases and allograft rejection (23, 24, 37). Tregs are critical for the induction of transplant tolerance (38, 39). Here we revealed that treatment with ESAT-6 increased FoxP3 expression in skin allografts as well as the frequency of CD4^+^FoxP3^+^ Tregs in LNs and spleen of recipient mice. We also found that depletion of Tregs using anti-CD25 Ab abolished the EAST-6-mediated extension of cardiac allograft survival. Here we acknowledge that CD25^+^ Treg-depleting Ab may also delete some of the conventional effector T cells, which is a limitation of this study confirming a role for Tregs in ESAT-6-induced allograft survival. However, it’s already well known that Tregs suppress alloimmunity. Furthermore, ESAT-6 also induced Tregs from CD4^+^CD25^-^ T cells in our study *in vitro*. Thus, our findings suggest that ESAT-6 inhibits allograft rejection by, at least in part, increasing CD4^+^FoxP3^+^ Tregs.

Interestingly, we found that administration of ESAT-6 did not significantly alter the maturation of DCs in the context of murine allotransplantation. ESAT-6 has previously been shown to regulate maturation/activation of DCs. It was found that ESAT-6 could bind to the surface receptors of DCs, including TLR2 (40) and TLR4 (41), resulting in a series of signal pathways that regulated the DC maturation and activation (42). In addition to its effects on DC maturation, ESAT-6 also increased the production of proinflammatory cytokines, including IL-6, TNF-α and IL-12, in DCs (41). Moreover, ESAT-6 promoted IL-6 and TGF-β secretion by DCs via activating TLR-2/MyD88 signaling pathway in mice infected by Mtb, thus enhancing Th17 cell responses (40). However, an earlier study revealed dual regulatory effects of ESAT-6 on DCs. In that study, human peripheral blood monocytes were used to induce immature DCs first and then mature DCs. The treatment with ESAT-6 inhibited DC maturation and activation, decreased IL-12, and increased the level of IL-23, thereby strengthening Th17 but impeding Th1 responsiveness (31). Therefore, the impacts of ESAT-6 on DCs may be complicated than originally thought. However, our findings showed that it did not alter DC maturation in the context of vigorous alloimmunity. Our *in vitro* data also demonstrated that ESAT-6 induced CD4^+^FoxP3^+^ Tregs and suppressed conventional T cell proliferation in the absence of DCs or APCs, indicating that it mainly works on Treg/T cells, but not DCs/APCs. On the other hand, suppression of conventional CD4^+^CD25^-^ T cell proliferation *in vitro* by ESAT-6 could be attributed to the simultaneous induction of Tregs that in turn inhibited the T cell proliferation.

The transcriptional factor c-Rel is a member of NF-ĸB/Rel family and governs the development of Treg cells by facilitating the formation of an enhanceosome specific for FoxP3 transcription (43) and regulating the synthesis of endogenous IL-2 (44). When intracellular IĸB molecule is phosphorylated upon stimulation, c-Rel translocates from the cytoplasm to the nucleus, which in turn induces the transcription of its downstream target genes (45). We found that ESAT-6 augmented both phosphorylation of IkBα and nuclear expression of c-Rel, while rapamycin or CFP-10 failed to do so, indicating that ESAT-6 induces CD4^+^Foxp3^+^ Tregs by specifically upregulating IĸBα/c-Rel signaling.

In this study, however, there were some limitations that need to be addressed in the future research. The prolongation of allograft rejection by ESAT-6 alone was moderate as all allografts were rejected within 30 days, although combined treatment with both ESAT-6 and rapamycin worked much better. Thus, future study should determine if higher doses or longer treatment would further prolong allograft survival. Moreover, it’s unknown whether the suppression of alloimmunity and induction of the Tregs are donor-specific. Studies on the allospecific effects of ESAT-6 are warranted in the future research. It’s also unclear if ESAT-6 works on the upstream signaling of IĸBα/c-Rel. Future research should determine whether ESAT-6 also affects the upstream signaling, including TLR/MyD88/TRAF6 axis, and what exact molecules ESAT-6 binds. Understanding of more mechanisms of its action would help design strategies to improve its efficacy in the suppression of allograft rejection. Finally, the future research may be focused on clinical trials using ESAT-6 protein to inhibit human allograft rejection.

In conclusion, ESAT-6 significantly prolonged both murine skin and heart allograft survival, alleviated CD3^+^ T cell infiltration and increased Foxp3^+^ Tregs in an allograft. ESAT-6 augmented the percentage of CD4^+^Foxp3^+^ Tregs, whereas it decreased the frequency of Th1 and CD4^+^/CD8^+^ effector T cells in both spleen and LNs post-transplantation. It also induced CD4^+^Foxp3^+^ Tregs from CD4^+^CD25^-^ T cells *in vitro* by activating IĸBα/c-Rel signaling pathway. Furthermore, it suppressed conventional T cell proliferation *in vitro*, with an increase in IL-10 and decrease in IFN-γ production. Thus, ESAT-6 suppresses allograft rejection by inducing CD4^+^Foxp3^+^ Tregs through IĸBα/c-Rel signaling pathway. These findings may be implicated for clinical transplantation.

## Data Availability

The datasets presented in this study can be found in online repositories. The names of the repository/repositories and accession number(s) can be found in the article/**Supplementary Material**.
