# Supplementary material for: ESAT-6 protein suppresses allograft rejection by inducing CD4+Foxp3+ regulatory T cells through IκBα/cRel pathway
Source: Front Immunol. 2025 Jan 9;15:1529226. doi: 10.3389/fimmu.2024.1529226 (PMC11754228; doi:10.3389/fimmu.2024.1529226)
Supplement: Supplementary file 1 [file DataSheet1.docx]

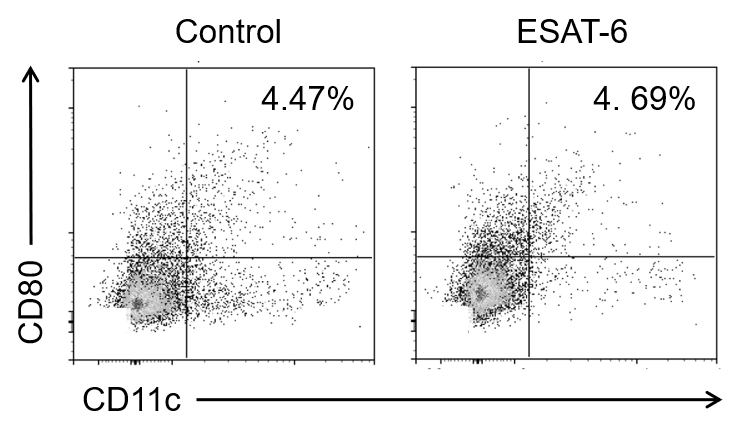

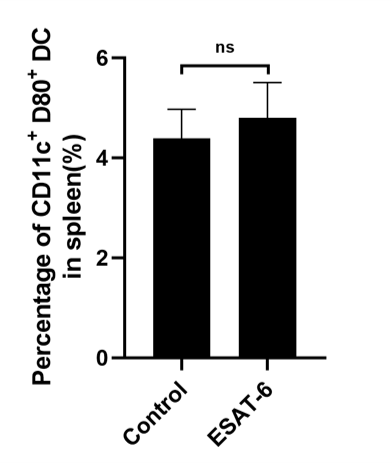

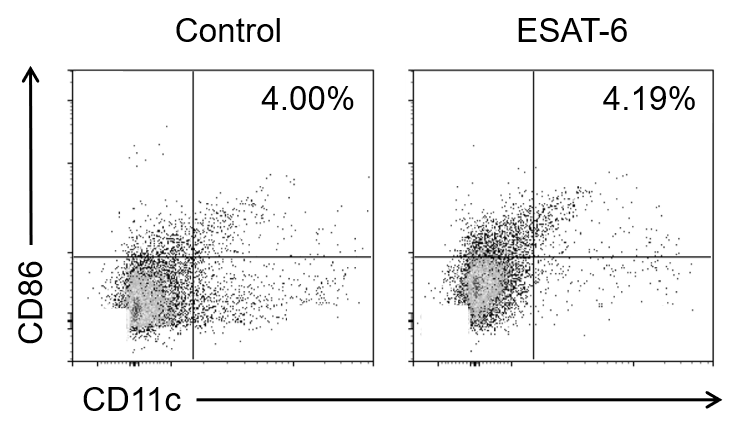

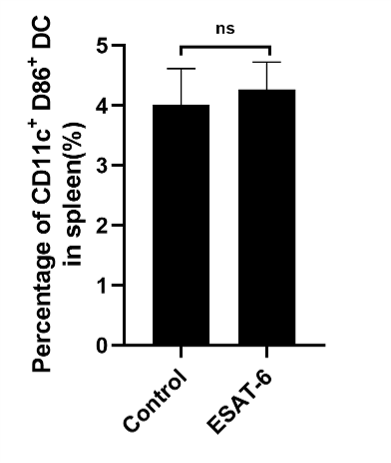


A

D

C

B

**Supplementary Figure S1. ESAT-6 does not change the percentage of CD11c^+^CD86^+^ and CD11c^+^CD80^+^ cells in the spleen of recipient mice.**

Spleen cells from B6 recipient mice treated with ESAT-6 were collected 10 days after transplantation with BALB/c skin, and analyzed using FACS. The representative dot plots of CD11c^+^CD86^+^ cells in spleen are presented **(A)**. Data of column graph show the average percentage of CD11c^+^CD86^+^ mature DCs **(B).** The representative dot plots of CD11c^+^CD80^+^ cells in spleen also are presented **(C)**, while data of column graph display the percentage of CD11c^+^CD80^+^ mature DCs **(D).** Data are shown as the mean ± SD (n = 5 mice/group, “ns”: non-significant). One of two separate experiments is shown.
